# Laparoscopic Repair of a Vesicovaginal Fistula After Total Abdominal Hysterectomy

**DOI:** 10.7759/cureus.78297

**Published:** 2025-01-31

**Authors:** Talal AlQattan, Reem AlMehzaa, Abida Qureshi, Saeed AlSary

**Affiliations:** 1 Surgery, Bahrain Defence Force Hospital, Riffa, BHR; 2 Obstetrics and Gynecology, Bahrain Defence Force Hospital, Riffa, BHR; 3 Obstetrics and Gynecology, Ministry of National Guard - Health Affairs, Riyadh, SAU; 4 Obstetrics and Gynecology, King Abdullah International Medical Research Center, Riyadh, SAU; 5 Obstetrics and Gynecology, King Saud Bin Abdulaziz University for Health Sciences, Riyadh, SAU

**Keywords:** laparoscopic gynecology surgery, laparoscopic repair, obstetrics & gynecology, total abdominal hysterectomy, urology surgery, vesicovaginal fistula

## Abstract

Several complications may arise postoperatively in gynecological surgeries including vesicovaginal fistula (VVF). VVF is an abnormal communication between the vagina and bladder which causes leakage of urine from the bladder into the vagina. It is associated with many etiologies such as sexual intercourse at an early age, prolonged/obstructed labor, malignancies, radiation therapy, and post-pelvic surgeries. Here, we report the case of a 50-year-old woman who developed a VVF after a total abdominal hysterectomy.

## Introduction

A vesicovaginal fistula (VVF) is an abnormal connection between the bladder and the vagina, resulting in a continuous, involuntary discharge of urine into the vaginal canal [1]. This condition can arise from various causes, including prolonged and obstructed labor, surgical complications, or trauma [1,2]. VVF not only leads to significant physical discomfort but also has profound social and emotional consequences for affected individuals. It remains a serious health issue, particularly in regions with limited access to timely obstetric care. Understanding its causes, symptoms, and treatment options is crucial for improving outcomes and restoring the quality of life for those affected. This case report aims to showcase how our patient presented, compare our approach to the management to other available articles with similar cases, and mention different treatment approaches that could have been used as alternatives such as conservative management, robotically, or laparoscopically.

## Case presentation

A 50-year-old female, para 2 with two previous abortions, underwent a total abdominal hysterectomy for fibroids and menorrhagia at a different hospital. She presented to the outpatient clinic in our hospital five months later complaining of continuous leakage of urine into the vagina. The patient developed symptoms of urine incontinence, loss of a desire to void, and absence of a bladder filling sensation a month after her surgery. As a result, she required diapers.

The patient presented to the clinic 14 days after a total abdominal hysterectomy complaining of a one-day history of watery vaginal discharge. She was diagnosed with VVF and underwent VVF repair two months later.

**Figure 1:**
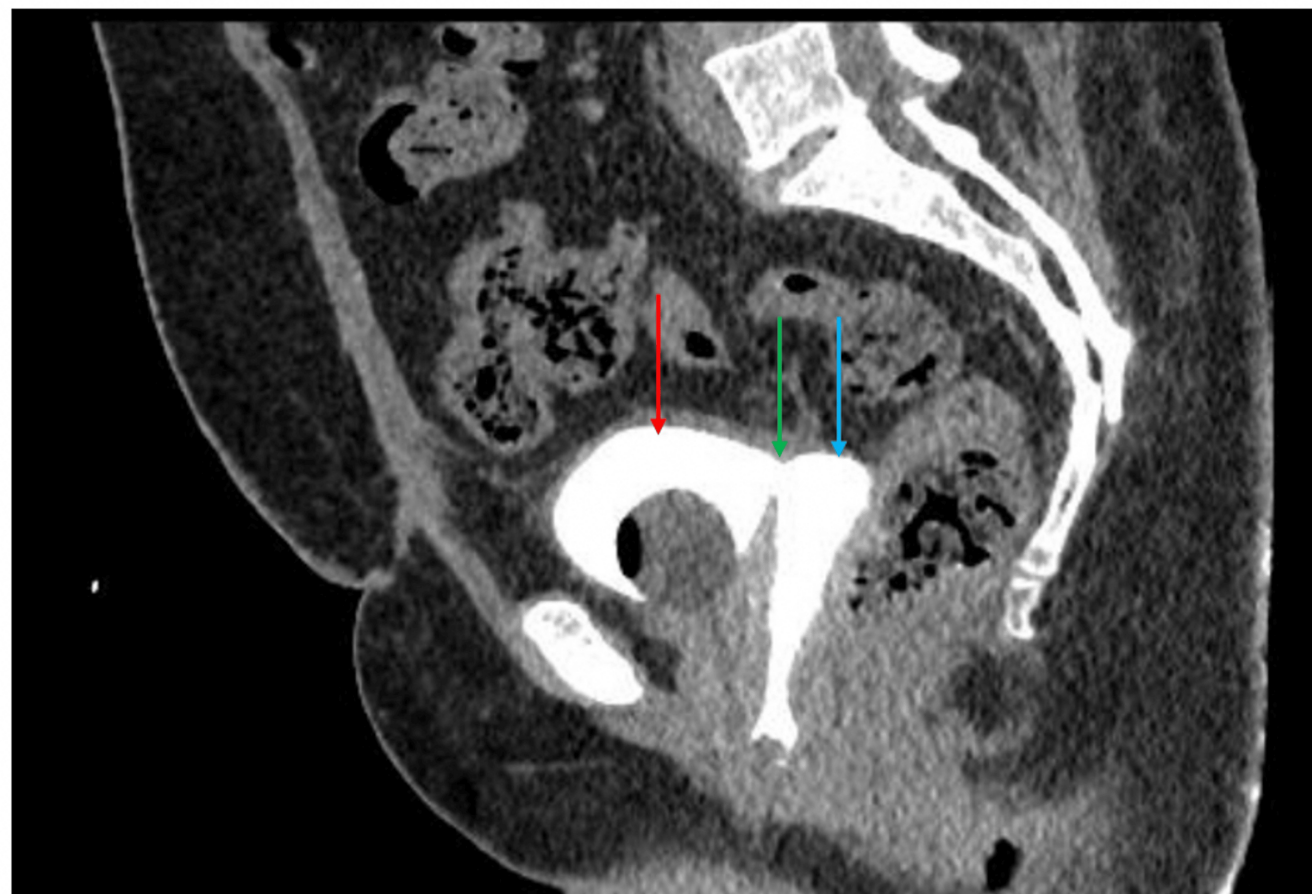
Preoperative CT cystogram with contrast. The red arrow is pointing at the bladder. The green arrow is pointing at the fistula. The blue arrow is pointing at the vagina.

Five months later, she presented to the outpatient clinic in our hospital with the same complaint. A urine culture was sent and a CT cystogram was ordered, and she was started on antibiotics until the end of her hospital stay. The CT cystogram showed a fistulous tract measuring around 7 × 4.5 mm between the superior aspect of the urinary bladder and vagina, as shown by the arrow in Figure 1. Two days after the CT cystogram, she was scheduled for a diagnostic cystoscopy and laparoscopy in light of the CT cystogram findings. Cystoscopy findings included a VVF measuring 6 × 5 mm 2-3 cm from the right ureter and 2 cm from the left ureter. Diagnostic laparoscopy findings included omentum adhesion onto the anterior abdominal wall mainly on the left side, bowel adherent to the left anterior abdominal wall, omentum densely adherent to the sigmoid, omentum adherent to the bladder, and bladder adherent to the pelvic wall.

During the diagnostic laparoscopy, Ligasure was used for adhesiolysis causing the release of omentum from the anterior abdominal wall. A malleable retractor was inserted into the vagina to assess the bladder laparoscopically. Dissection between the bladder and vaginal space was done, where some edematous tissue was noticed. The bladder was opened as low as possible and the fistula was visualized. Fibrotic tissue around the bladder and vagina was noticed and mobilization of the bladder from the vagina was done. A second fistulous tract was discovered next to the first one, which was trimmed and sutured with V-lock 2-0 sutures after visualizing the ureters. The malleable retractor was removed. Before reinforcing the sutures, methylene blue was injected. As minimal leak was noted, the sutures were placed at the site of the leak. Correspondingly the bladder suspension was released, cystoscopy was performed, the bladder was flushed, and the suture line was intact indicating no leakage. The laparoscope was reintroduced and no peritoneal leakage was noted.

Postoperatively, her vitals were monitored. The initial plan was to keep her on intravenous fluids, paracetamol (a painkiller), ciprofloxacin (the antibiotic of choice), solifenacin (an anticholinergic) for 30 days to prevent bladder spasms. A Foley’s catheter was inserted and advised to be kept for 24 days. On the second postoperative day, both peptic ulcer disease and deep vein thrombosis prophylaxis were initiated, and ciprofloxacin was switched to nitrofurantoin based on the mid-stream urine.

**Figure 2:**
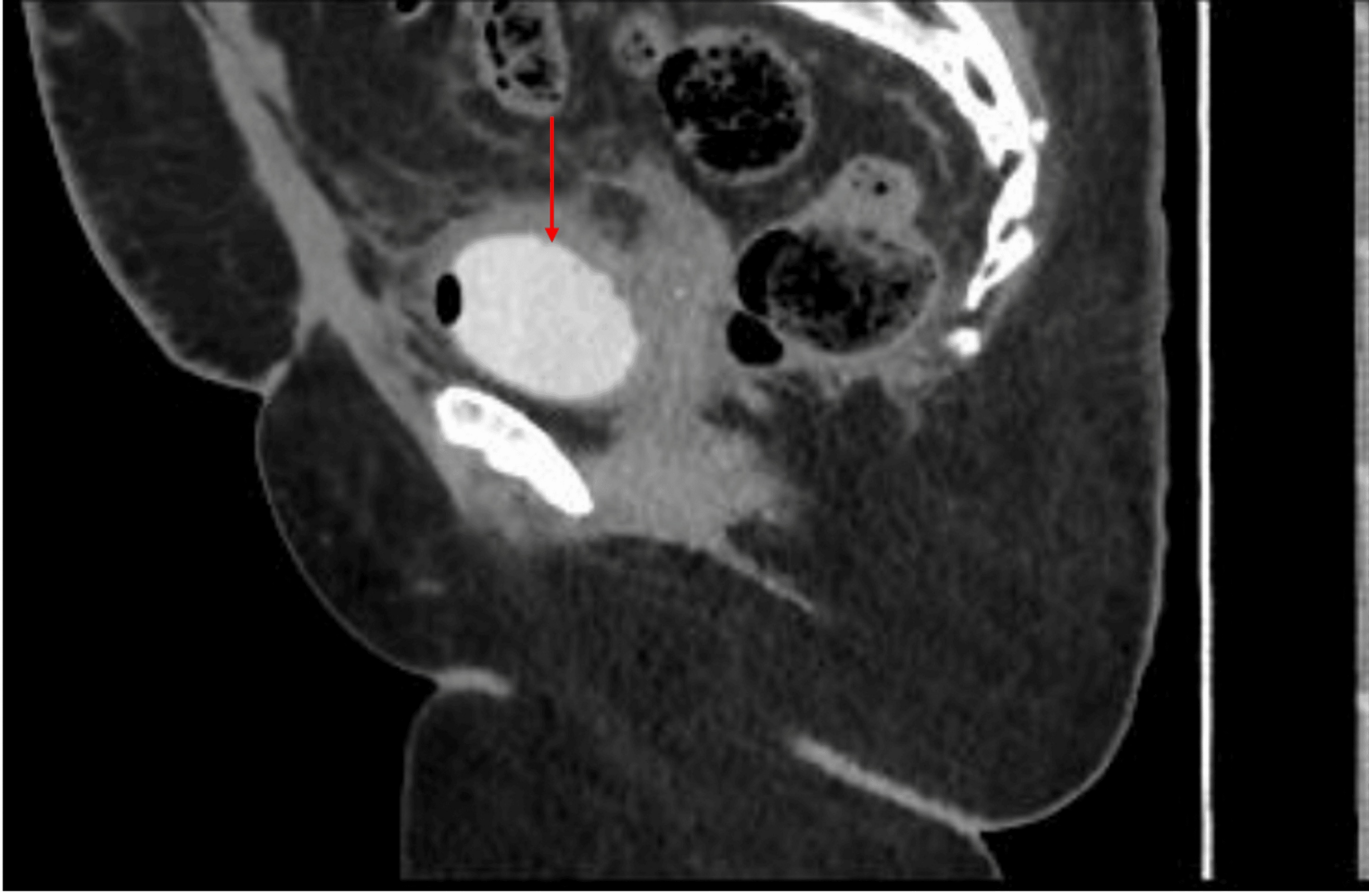
Postoperative CT cystogram with contrast. The red arrow is pointing at the bladder.

Upon discharge, she was doing well, vitally stable with no active complaints. She was advised to continue taking oral nitrofurantoin for 24 days, oral solifenacin for 30 days days, and paracetamol for 14 days. Regular follow-ups were done. One week after discharge, mid-stream urine was negative. Three weeks postoperatively, a CT cystogram was repeated and there was no evidence of contrast leakage into the vagina, as shown by the arrow in Figure 2. Within three weeks, the patient started complaining of vulval irritation which was managed with povidone-iodine (betadine) vaginal pessary, and a combination of symptoms of hot flushes, night sweats, pelvic pain, and dysuria, which were managed with hormonal replacement therapy. Follow-up appointments in the clinic were scheduled. During these appointments, the patient did not have any complaints.

## Discussion

VVF is considered to be rare in the developed world. Hysterectomy is the most common gynecological surgery that results in VVF [1,3]. There are many different surgical approaches depending on the surgeon. Although laparoscopic procedures have the highest rate of fistula formation and the transvaginal approach has the lowest rate, no approach is superior to the other [4,5]. Whether it was done open, transabdominally, laparoscopically, or robotically, some studies have shown that the overall success rate is 87.5%, regardless of the approach [1]. In comparison to conservative treatment, conservative measures have a higher tendency to fail. The transabdominal approach usually has a prolonged hospitalization stay in comparison to laparoscopic and robotic approaches, which is a major benefit of these two approaches [5].

The most common complication of fistula repair is recurrence of the fistula. To prevent a recurrence, omental flaps could be considered to reduce the rate of failure and recurrence [2,6]. However, it is not routinely used. There has been a recent use of fibrin glue as an alternative to omental flaps. A comparison study between the two showed results significantly in favor of fibrin glue. It is recommended that the fibrin glue should be used when laparoscopic repair is done [6].

The diagnosis of VVF is usually made with the complaint of continuous urine leaking through the vagina, usually one to two weeks postoperatively after gynecological or pelvic surgery [1]. Our patient presented 14 days postoperatively, which is within the same duration where the complication would be expected to arise.

Insertion of a Foley catheter is crucial for continuous drainage of the bladder postoperatively. The patient is advised to be catheterized for around two to three weeks, or in our case 24 days [2]. Anticholinergics (solifenacin used in our patients) are given to patients who have bladder spasms but might compromise healing and cause pain [1,2]. It is used to prevent complications of the repair such as urgency and stress urinary incontinence. Antibiotic coverage is also required until the removal of the catheter as a prophylactic measure; in this case, nitrofurantoin was used. The combination of all these management approaches contributes to the overall prevention of postoperative complications, most commonly recurrent fistula formation.

## Conclusions

The laparoscopic repair of VVF following a total abdominal hysterectomy presents a promising and effective approach to managing this complex condition. With advancements in minimally invasive surgical techniques, patients benefit from reduced recovery times, less postoperative pain, and improved cosmetic outcomes compared to traditional open surgery. As VVF can significantly impact a woman’s quality of life, timely and effective intervention is critical. Continued research and training in laparoscopic methods will enhance surgical outcomes and broaden access to this vital treatment option. By integrating laparoscopic repair into standard care protocols for VVF, we can improve the overall management of women affected by this condition, promoting their physical health and emotional well-being. Moving forward, a commitment to educating healthcare professionals and raising awareness about VVF will be essential in ensuring that all women receive optimal care.
